# Identification of Mortalin as the Main Interactor of Mycalin A, a Poly-Brominated C-15 Acetogenin Sponge Metabolite, by MS-Based Proteomics

**DOI:** 10.3390/md22020052

**Published:** 2024-01-23

**Authors:** Elva Morretta, Alessandra Capuano, Gilda D’Urso, Antonia Voli, Matteo Mozzicafreddo, Sonia Di Gaetano, Domenica Capasso, Marina Sala, Maria Carmina Scala, Pietro Campiglia, Vincenzo Piccialli, Agostino Casapullo

**Affiliations:** 1 Department of Pharmacy, University of Salerno, 84084 Fisciano, Italy; emorretta@unisa.it (E.M.); acapuano@unisa.it (A.C.); gidurso@unisa.it (G.D.); avoli@unisa.it (A.V.); msala@unisa.it (M.S.); mscala@unisa.it (M.C.S.); pcampiglia@unisa.it (P.C.); 2 PhD Program in Drug Discovery and Development, University of Salerno, 84084 Fisciano, Italy; 3 Department of Clinical and Molecular Sciences, Marche Polytechnic University, 60126 Ancona, Italy; m.mozzicafreddo@univpm.it; 4 Institute of Biostructures and Bioimaging, Consiglio Nazionale delle Ricerche, Via Pietro Castellino 111, 80131 Napoli, Italy; sonia.digaetano@cnr.it; 5 Department of Physics, Ettore Pancini, University of Naples Federico II, Via Cintia 21, 80126 Naples, Italy; domenica.capasso@unina.it; 6 Department of Chemical Sciences, University of Naples Federico II, Via Cintia 21, 80126 Naples, Italy

**Keywords:** proteomics, drug affinity responsive target stability, targeted-limited proteolysis, multiple reaction monitoring, marine antitumoral compound, molecular docking, heat shock proteins

## Abstract

Mycalin A (MA) is a polybrominated C-15 acetogenin isolated from the marine sponge *Mycale rotalis*. Since this substance displays a strong antiproliferative bioactivity towards some tumour cells, we have now directed our studies towards the elucidation of the MA interactome through functional proteomic approaches, (DARTS and t-LIP-MS). DARTS experiments were performed on Hela cell lysates with the purpose of identifying MA main target protein(s); t-LiP-MS was then applied for an in-depth investigation of the MA–target protein interaction. Both these techniques exploit limited proteolysis coupled with MS analysis. To corroborate LiP data, molecular docking studies were performed on the complexes. Finally, biological and SPR analysis were conducted to explore the effect of the binding. Mortalin (GRP75) was identified as the MA’s main interactor. This protein belongs to the Hsp70 family and has garnered significant attention due to its involvement in certain forms of cancer. Specifically, its overexpression in cancer cells appears to hinder the pro-apoptotic function of p53, one of its client proteins, because it becomes sequestered in the cytoplasm. Our research, therefore, has been focused on the possibility that MA might prevent this sequestration, promoting the re-localization of p53 to the nucleus and facilitating the apoptosis of tumor cells.

## 1. Introduction

The marine ecosystem is a remarkable source of secondary metabolites, characterized by a wide structural diversity and mostly produced by several species as a protection against their predators. Thanks to their bioactivity profiles, these compounds are invaluable in drug discovery efforts and serve as a foundation for developing new drugs to treat various human diseases [1,2,3].

**Figure 1:**
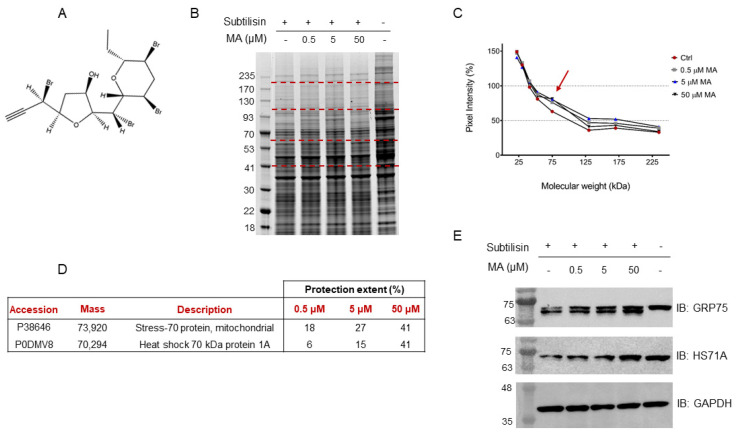
(**A**) Mycalin structure. (**B**) Coomassie stained gel showing proteins protection from subtilisin upon MA interaction. Dashed red lines indicate gel slices submitted to in situ tryptic digestion. Molecular weights alongside the ladder are expressed in kDa. (**C**) Densitometric analysis of the SDS-PAGE in panel (**B**), performed through ImageJ and reporting the pixel intensity of each gel region vs. its corresponding molecular weight range. Positive control intensities, rated at 100%, are not reported. As shown by the red arrow, the lowest MA concentration induces the highest pixel intensity increase around 75 kDa, where the protection is also dependent on the molecule amounts. (**D**) Mortalin and HS71A protection extent (%) calculated from the Mascot matches, as follows: [(Matches_MA_ − Matches_Ctrl_)/Matches_Lysate_] × 100. The reported values are the results of two DARTS biological replicates. (**E**) Immunoblotting analysis with anti-mortalin (i.e., anti-GRP75) and anti-HS71A antibodies. GAPDH has been used as a loading normalizer.

Halogenated acetogenins found in marine environments, originating from the polyketide pathway, are plentiful in red algae, notably within the *Laurencia* genus. Among these compounds, some exhibit unique structural features and hold promise for pharmacological applications. Mycalin A (MA) (Figure 1A), a polybrominated C-15 acetogenin of marine origin isolated from the sponge *Mycale rotalis* [4,5], possesses noteworthy antiproliferative properties against human melanoma (i.e., A375) and cervical adenocarcinoma (i.e., HeLa) cells. Its mode of action involves the induction of apoptosis, a programmed cell death mechanism [6,7].

A rational approach to the drug discovery and development pipeline involves the knowledge of the interacting profile of a drug candidate, for a comprehensive evaluation of its poly-pharmacological activities and potential side effects. To this end, mass spectrometry-based chemical proteomics stands for a state-of-the-art strategy to select the specific target(s) and off-target(s) of interesting small bioactive molecules, in pseudo-physiological conditions. In the classical fishing-for-partners approach, protein targets of a small molecule (SM) are selected among a complex protein mixture, such as a cell lysate, on the basis of their affinity with the compound, which was previously covalently linked to an opportune tag (or a solid support) [8]. Recently proposed procedures aim to work with unmodified SMs, in a more general and versatile way. Such approaches are likely designed to preserve SMs natural properties and bioactivity, which could be otherwise affected by any type of covalent modification [8,9].

Functional proteomic techniques based on untargeted (or targeted) limited proteolysis coupled with mass spectrometry can be very helpful to solve molecular recognition events between a SM and a protein. These techniques focus on how the interaction affects protein conformation and stability [10].

DARTS (Drug Affinity Responsive Target Stability) is a functional proteomic approach that relies on the stabilization of a protein upon specific binding with a SM [11,12,13]. Targeted limited proteolysis coupled to mass spectrometry (t-LiP-MS) is another approach that operates on the same DARTS principles and is particularly useful for mapping the binding regions of a specific protein of interest with its SM partner [14,15,16].

These techniques are valuable tools for understanding the molecular mechanisms underlying protein–SM interactions. They can provide insights into how binding events affect protein structure and function, which is essential in drug discovery and target identification.

On this basis, we decided to combine the information coming from DARTS and t-LiP-MS to disclose the target profile of MA. This analysis identified mortalin, a mitochondrial Hsp70 also known as GRP75, and HS71A as its main targets, also shedding light on the binding features of their complexes.

Mortalin plays a critical role in the regulation of protein folding and quality control and is also involved in mitochondrial homeostasis. Interestingly, a fraction of mortalin is also found in the cytosol, where it interacts with proteins involved in signaling, apoptosis, or senescence. In addition to cytosolic Hsp70s, whose expression are significantly elevated in different type of cancers (e.g., colon cancer, gastric cancer, breast cancer, ovarian cancer, hepatocellular carcinoma, and prostate cancer) [17], mortalin plays an anti-apoptotic role in cancer. The close interaction between the p53 protein, known for its pivotal role in tumor prevention, and mortalin, a key regulator of cellular stress response, unveils intriguing complexity in cancer processes. The ability of p53 to orchestrate apoptosis, or programmed cell death, is modulated by its interaction with mortalin, which appears to play an inhibitory role, influencing the sensitivity of cells to apoptosis. In situations of cellular stress, elevated expression of mortalin may act as a brake on p53 ability to eliminate damaged cells, thereby favoring cell proliferation and contributing to tumor development. This intricate relationship between p53 and mortalin opens avenues for new therapeutic perspectives. Consequently, mortalin is considered a promising target in the control of several human pathologies [18,19].

Based on the relevance of this protein, further analyses on the interactions at a molecular level between MA and mortalin were performed, utilizing molecular docking and surface plasmon resonance (SPR). Additionally, the biological role of MA was also investigated.

## 2. Results

DARTS and t-LiP-MS approaches were applied and optimized to give a comprehensive characterization of MA interactome. The experimental design presented here is based on five main steps, consisting of: (a) application of a DARTS-MS protocol for the characterization of MA interactome and the selection of its main protein partner(s); (b) DARTS-MS data validation through Western Blotting analysis; (c) application of t-LiP MS procedure to investigate the interaction features between MA and its main target(s) [20], later deepened through (d) molecular docking and SPR, along with (e) the conduction of in vitro/in vivo assays to evaluate MA biological properties.

### 2.1. Identification of Mycalin A Cellular Target(s) through DARTS

DARTS exploits the thermodynamic stabilization conferred by a molecule to its protein target(s). Indeed, under physiological conditions, multiple alternative conformations of a protein are in equilibrium with each other, but the interaction with a specific ligand highly favors the molecule-bound conformation. This phenomenon leads to a thermodynamically more stable state in which the target protein(s) conformational fluctuations are radically decreased, thus strikingly increasing resistance to limited proteolysis [11,12,13]. Subsequently, separation and visualization of the differentially treated protein mixtures (i.e., incubated or not with the compound) by 1D-SDS–PAGE gives evidence of the proteins with altered proteolytic patterns in presence of the binding molecule: the intensity of their corresponding gel bands is higher in the compound-treated samples compared to the control ones. Thus, target proteins can be subsequently identified through classical bottom-up proteomics [12,20,21,22].

In this scenario, a HeLa cell lysate was incubated with either MA (0.5 μM, 5 μM or 50 μM final concentrations), to allow the interaction of this substance with its partner(s), or DMSO as the control. Each sample was then submitted to limited proteolysis, in native conditions, with the protease subtilisin, keeping an undigested DMSO-treated sample as a positive control. Subtilisin was quenched and the samples were separated through 1D-SDS-PAGE and visualized through Coomassie staining (Figure 1B). The mycalin A interacting proteins were thus recognized both by visually inspecting the stained gel and by performing an accurate densitometric analysis (Figure 1C): the gel bands whose intensity increased at increasing MA concentrations, in respect to the negative control (i.e., DMSO and subtilisin treated sample), were carefully excised (red dashed lines, Figure 1B) and digested as previously reported [23]. Subsequently, nano-ESI-UHPLC-MS/MS analysis of the digested samples followed by Mascot database search gave protein identification, allowing to then perform a semi-quantitative analysis to point out MA interacting proteins, as previously reported [20].

More in details, these proteins were identified by directly comparing the Mascot matches with those of the positive control experiment (i.e., DMSO-treated, and undigested sample), used as a normalizing factor to correct for proteins different abundance in the lysate, and of the negative control one, which values were an indication of the protein’s maximal response to the protease. Thus, a protection percentage was calculated for each protein identified in correspondence to its molecular weight. To obtain a more confident list of putative MA binding proteins, this experiment was carried out in duplicate.

Based on the Mascot parameters (Table S1), the mitochondrial Stress-70 protein mortalin (i.e., GRP75) was identified as one of the most reliable MA interacting partners, since it was protected in both experiments showing direct proportionality between the protection extent and MA concentrations. Additionally, the cytosolic Heat shock 70 kDa protein 1A (i.e., HS71A), even if with a lower protection level in one of the two replicates, was also identified as MA protein partner (Figure 1D).

The DARTS-MS results were subsequently validated through Western Blotting, using specific antibodies for the Hsp70 isoforms. As can be observed in Figure 1E, increasing MA concentrations clearly protect both full-length mortalin and HS71A from subtilisin, compared to the control sample (first lane of the membrane), in which less intact proteins can be detected. Moreover, looking at mortalin WB analysis (Figure 1E, first row), the signal at higher molecular weight, corresponding to the full-length protein, comes with another signal due to a fragment produced by the removal of a small N-terminal peptide by subtilisin (antibody epitope: residues 525–679). This fragment also appears to be protected from proteolysis. The extent of subtilisin protection induced by MA on the two targets was also analyzed through a densitometric analysis of the Western Blot’s full-length Hsp70s, using Glyceraldehyde 3-phosphate dehydrogenase (i.e., GAPDH) as a loading normalizer (Figure S1).

### 2.2. Hsp70 Isoforms

The intricate balance of processes encompassing protein synthesis, folding, trafficking, assembly, and degradation, known as proteostasis or protein homeostasis, is a crucial mechanism for proper cell functioning. The highly conserved and ubiquitous molecular chaperones belonging to the 70 kDa heat shock protein (Hsp70) family play pivotal roles in maintaining protein homeostasis. They are essential in conditions associated with cellular stress but also in optimal growth environments. Members of the Hsp70 family are engaged in a wide array of essential cellular tasks. These include facilitating the folding of newly synthesized proteins, assisting the translocation of polypeptides into mitochondria and the endoplasmic reticulum, disassembling protein complexes, and modulating protein activity. Hsp70 proteins play a crucial role in preventing the aggregation of proteins and facilitating the refolding of misfolded or denatured proteins. They also act to solubilize aggregated proteins and collaborate with cellular degradation machinery, such as the proteasome and autophagy pathways, to eliminate abnormal proteins and aggregates. Additionally, Hsp70s are involved in the regulation of the heat shock response and play a role in controlling apoptosis [24].

Mortalin is found to be overexpressed in many types of tumor tissues [25], a fact that points out its significant involvement in oncogenesis. One of its primary oncogenic functions lies in its ability to bind the tumor suppressor p53, sequestering it in the cytoplasm. As a result of this binding, p53 cannot transit into the nucleus and carry out its role as a transcription factor for some genes, such as p21 and puma, responsible for cell cycle arrest and apoptosis in cancer cells [26].

The structure of mortalin includes two major domains: a 42-kDa N-terminal nucleotide binding domain (NBD), also known as the ATPase domain, and a 25-kDa C-terminal substrate binding domain (SBD), or peptide binding domain (PBD).

The NBD has four different subdomains (IA, IB, IIA, and IIB), which come together to shape the nucleotide-binding pocket. This region exhibits a conformational adaptability upon nucleotide occupancy and interaction with co-chaperone proteins. Indeed, the allosteric ATP binding and its hydrolysis regulate its chaperoning activities. In addition to its interactions with ATP/ADP and co-chaperones, in this region was also mapped the binding site of MKT-077 (residues 252–310), a water-soluble rhodocyanine dye analog well-known as a mortalin inhibitor, which can modulate the sequestration of p53 [27]. MKT-077 is located between mortalin residues 267–271 and in proximity to the side chain of Y196, inducing alterations in the conformation of the interacting mortalin β-strand, which is comprised of residues 260–270. Simultaneously, as the loop containing Y196 is expected to come into close contact with residues 267–271 within mortalin’s ATP-bound conformation, MKT-077 might also contribute to the destabilization of this ATP-bound state. This induced destabilization, in a cascading manner, indirectly hampers the association between mortalin and Tid1, a mitochondrial co-chaperone. Consequently, MKT-077 presents several potential mechanisms to hinder mortalin’s general functionality and, more specifically, to obstruct the interaction between mortalin and p53 [27].

The substrate-binding domain (SBD) of mortalin is constructed from two sets of four-stranded anti-parallel β-sheets. Within this domain, there is a 10-kDa substrate lid that envelops and seals substrates when mortalin is in the ADP-bound state. Remarkably, this domain exhibits a preference for binding to peptides with aliphatic and basic residues. These residues are typically buried deep within the α-strands of properly folded proteins but become exposed when the protein is misfolded.

### 2.3. Analysis of Mycalin A Interaction Features with Mortalin and HS71A through t-LiP-MS

To deepen the investigation on MA interaction with both mortalin and HS71A, a t-LiP-MS strategy has been optimized.

T-LiP-MS is a recently developed method [16] that enables probing proteins conformational changes in complex biological environments (e.g., in cell lysates neither pre-fractionated nor enriched for a given protein) by coupling limited proteolysis (LiP) with Multiple Reaction Monitoring (MRM) mass spectrometry [14,15]. This technique gives information on the interactions between a SM and its target, pinpointing the protein regions involved in the binding. These regions that undergo structural changes in consequence of the ligand recognition can be identified through a quantitation of their tryptic peptides.

In a typical t-LiP-MS experiment, a cell lysate is obtained in native conditions, incubated with the molecule of interest (or just with the vehicle, as a control) and then submitted to a double in solution digestion protocol. At first, limited proteolysis in native conditions and with a broad-specificity protease (e.g., subtilisin) is performed, so that the target protein(s) initial cleavage sites are dependent on its structural features [10]. Subsequently, the protease is quenched, samples are shifted to denaturing conditions and an extensive tryptic digestion is carried out to generate peptide mixtures suitable for the MRM-MS analysis, devoted to the quantitation of the maximum number of fully tryptic peptides for the target protein(s).

Indeed, when trypsin is added to partially digested samples, its cleavages can either occur on the subtilisin products or in undigested proteins regions: in the former case, semi-tryptic peptides are produced alongside the fully tryptic ones, whereas in the latter circumstance only the fully tryptic peptides are generated. Since the interaction with a small molecule stabilizes a protein, inducing structural changes that might reduce subtilisin action onto the directly/distally involved regions, a larger amount of fully tryptic peptides will be observed in the compound-treated sample in respect of the untreated one. On the other hand, no significant change should be detectable for fully tryptic peptides corresponding to protein regions not involved in the interaction. Thus, fully tryptic peptides relative abundances differences are indicative of the protein structural changes, due to ligand binding.

To optimize our t-LiP-MS strategy, an in silico search was performed to build-up the MRM methods. At first, mortalin and HS71A fully tryptic peptides that are easily detectable by mass spectrometry were identified through the data repository Peptide Atlas [28], and then their best daughter ions were selected through SRM Atlas [29], searching fragments providing the highest intensity and lowest level of interfering signals. The obtained methods, comprising the three best daughter ions for each precursor, were then refined by testing them on a HeLa tryptic digest, to assign the peptides their correct retention times and select only the best transition for each one of them (Table S2).

Subsequently, HeLa lysate aliquots were incubated with MA (50 μM final concentration) or DMSO as the vehicle control. Each sample was then submitted to limited proteolysis, in native conditions, with the protease subtilisin, keeping an undigested DMSO-treated sample as a positive control. Subtilisin was then quenched, and the samples shifted to denaturing conditions and submitted to an extensive tryptic digestion and desalting. The obtained peptides mixtures were analyzed through UHPLC-MRM-MS on a QTRAP 6500 mass spectrometer. At first, subtilisin processed peptides were selected as the ones whose negative control sample intensities were lower than the 70% of the corresponding positive control ones (Table S2 and Figure S2). Subsequently, the comparison of these peptides between MA treated and untreated samples revealed the ones protected from subtilisin proteolysis as those having a higher intensity in the MA exposed sample, compared to the negative control. Thus, peptides whose fold changes were higher than 1.5 and whose related *p*-values were smaller than 0.05 were selected as symptomatic of the molecule protection on specific mortalin and HS71A regions, being either directly or distally involved in the interaction event.

**Figure 2:**
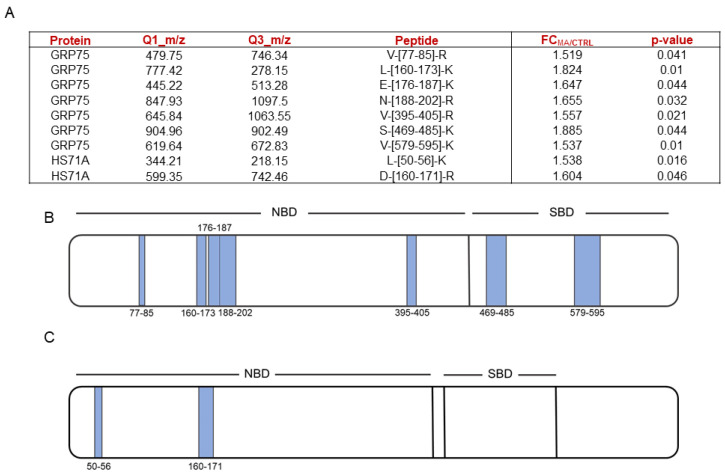
(**A**) MA-protected mortalin (i.e., GRP75) and HS71A fully tryptic peptides, reported with their Q1 and Q3 *m/z* values, the calculated MA over negative control fold changes and the corresponding *p*-values. Fold changes were calculated over the mean of peptides areas coming from three replicates. Schematic mortalin (i.e., GRP75, (**B**)) and HS71A (**C**) cartoons reporting the proteins NBDs and SBDs. T-LiP-MS protected peptides are represented as light blue bars, whose thickness is directly proportional to their length.

Thus, Figure 2A shows the selected peptides, reported with their fold changes and the relative *p*-values, whereas Figure 2B,C respectively show the same peptides represented on both mortalin and HS71A schematic cartoons. As can be observed, MA-protected peptides mapping for the highly conserved N-terminal nucleotide-binding domain (NBD) were identified for both Hsp70 isoforms, whereas peptides mapping for the substrate binding domain (SDB) could be identified only for mortalin.

HSP70’s 3D structures (Figure S3) gave evidence on the proximity of mortalin protected peptides, both in the NBD and SBD, while HS71A NBD protected peptides were not close to each other but faced the same α-helix.

### 2.4. Molecular Docking Analysis of Mycalin A Complexes with Mortalin and HS71A

In parallel to t-LiP-MS, a molecular docking analysis was performed to evaluate MA’s best interaction poses with both mortalin and HS71A NBDs and SBDs. Thus, the equilibrium dissociation constants (i.e., K_D,pred_) were calculated to predict the affinities of the complexes, and optimal geometries along with representations of the interactions were determined.

The predicted complexes between the Hsp70 isoforms and MA exhibited affinities in the micromolar/sub-micromolar range.

**Figure 3:**
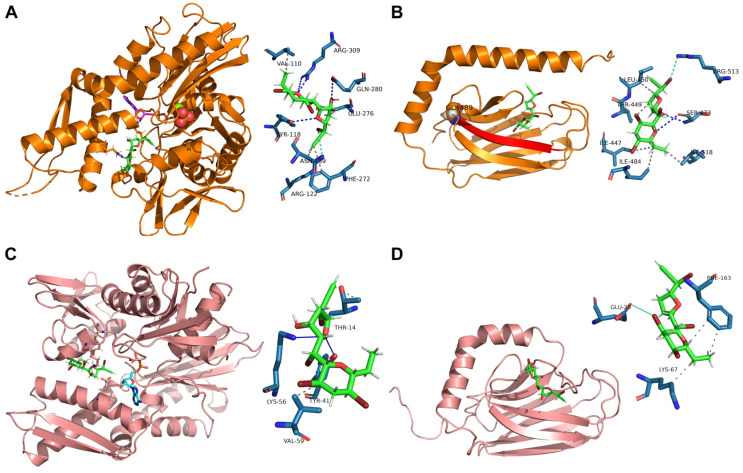
MA best predicted interaction poses with mortalin NBD (**A**) and SBD (**B**) and with HS71A NBD (**C**) and SBD (**D**) obtained through molecular docking. MA is represented through green sticks in all of the panels, whereas ADP is represented in purple stick in panel (**A**) (where the third phosphate is also shown as orange/red spheres and Mg^2+^ as a light green sphere) and in light blue stick in panel (**C**). MA interacting amino acidic residues are depicted in the zoomed schemes, with hydrophobic interactions, hydrogen bonds and halogen bonds shown in gray, blue and light blue lines, or dotted lines, respectively.

MA showed to bind mortalin NBD (K_D,pred_ of 5.254 ± 1.652 μM) very close to the ADP binding site, interacting with Val110, Tyr118, Arg122, Asn139, Phe272, Glu276, Gln280 and Arg309 (Figure 3A). Nevertheless, it also binds mortalin SBD with a higher affinity (K_d_,_pred_ of 4.456 ± 1.158 μM), forming an H-bond with Ser473, a halogen bond with Arg513, and five hydrophobic interactions with Ile447, Thr449, Leu450, Ile484, and Ile518. As shown in Figure 3B, Ile484 lies in the beta strand (represented in red) including Gly489 (shown as spheres).

Furthermore, as already reported for mortalin, MA showed to bind HS71A NBD (K_D,pred_ of 4.719 ± 1.231 μM), very close to the ADP binding site, interacting with Thr14, Tyr41, Lys56 and Val59 (Figure 3C). This finding was not surprising, given the well-known NDBs conservation across all the Hsp70s isoforms. Nevertheless, though in contrast with its mitochondrial counterpart, MA displayed only a halogen interaction with Glu20 and two hydrophobic interactions with Lys67 and Phe163 of HS71A SBD, explaining its lower affinity for this site (Figure 3D, K_D,pred_ of 27.95 ± 1.026 μM).

Comparing the t-LiP-MS with the molecular docking data, a very satisfactory correspondence could be observed. In detail, t-LiP-MS peptides that were identified as being protected by MA include amino acids that were involved in the best interaction poses engaged by the molecule with both mortalin SBD (Ser473 and Ile484 in S-[469-485]-K) and HS71A NBD (Lys56 in L-[50-56]-K), thus confirming the t-LiP-MS driven hypothesis of MA inducing structural changes in these Hsp70s regions. Regarding mortalin NBD, peptides including the amino acids involved in the best interaction poses with MA were not mapped in our t-LiP-MS analysis (Table S2) and thus not assayed, meaning no direct correspondence between the two experimental procedures was possible. Nevertheless, we were able to identify a consistent number of MA-protected peptides mapping for mortalin PO_4_^3−^/Mg^2+^ interacting region, right in the ADP binding site, which is juxtaposed to the molecular docking highlighted putative interaction region. Furthermore, the t-LiP-MS identified N-[188-202]-R peptide directly faces the α-helix comprising the molecular docking identified amino acidic residues. Thus, it is possible that our t-LiP-MS data reflected a long-range protective effect, due to a middle term conformational variation induced by MA.

### 2.5. MA Effects on p53 Transcriptional Activity

Since t-LiP-MS and molecular docking structural results pointed towards the same direction, especially for mortalin, we investigated if the MA–mortalin interactions could have a significant biological role, also giving a rationalization of the reported selective cytotoxic effects of MA. p53 is one of the most interesting mortalin client proteins, since it is strictly implicated in the mechanisms of cell death and apoptosis. Thus, we turned our attention to a potential modulation on the mortalin–p53 complex assembly mediated by MA, and its consequent result on different cell lines.

**Figure 4:**
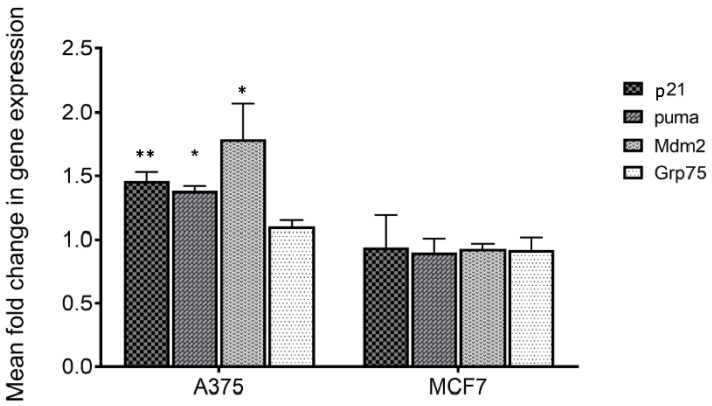
Histogram for data shown in Table 1. * *p* < 0.05; ** *p* < 0.01.

To verify if treatment with MA of responsive (A375) and not responsive cells (MCF7) [6] could act perturbing the mortalin–p53 interaction with the consequent releasing of p53 in the cytosol, the transcriptional activity of the latter was analyzed. For this purpose, p53 target genes including p21, mdm2 and puma were considered, and their expression was monitored by q-PCR. Mortalin expression levels were also analyzed. The obtained results show that all p53 target genes are overexpressed following treatment on responsive cells (Table 1 and Figure 4). Differently, no variation in expression of these genes was highlighted in MCF7 cells, in which MA did not have an anti-proliferative effect. Moreover, the expression of mortalin was not influenced by this treatment in both the analyzed cell lines. These data made us assume that MA treatment increases the activation of genes that are under p53 control, and indirectly demonstrate that the MA mechanism of action involves the dissociation of the mortalin–p53 complex, leaving p53 free to act as a transcriptional activator. It is worth noting that the results also confirmed the reported selectivity of action of MA towards specific cancer cells, a very important tool in the development of new cancer drugs.

### 2.6. Surface Plasmon Resonance Analysis of Mycalin A on the Mortalin–p53 Complex 

**Figure 5:**
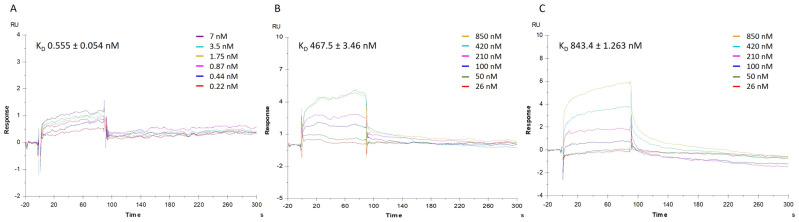
SPR analysis. Sensorgrams to evaluate (**A**) the binding affinity between p53 protein and mortalin (mortalin concentrations were 0.22, 0.44, 0.87, 1.75, 3.5, and 7 nM); and the competition between (**B**) mortalin-MA and (**C**) mortalin-MKT077. Concentration of MA or MKT077 were 26, 50, 100, 210, 420, and 850 nM while concentration of mortalin remained 13 nM. The equilibrium dissociation constants (K_D_) were derived from the ratio between kinetic dissociation (koff) and association (kon) constants. Experiments were repeated independently three times. Reported K_D_ is the mean ± SD of three independent experiments.

To corroborate qPCR results, which pointed towards a disturbance of mortalin–p53 interaction induced by MA, a complementary SPR approach was applied on a Biacore T200 instrument, to first measure the direct binding of mortalin with p53, conveniently immobilized on a CM5 sensor chip, to then perform competition assays. Thus, in the first instance, the affinity of mortalin for p53 protein was determined by injecting a series of increasing concentrations of mortalin (0.22–7 nM) onto the p53-immobilized chip. The mathematical manipulations and fitting operations, performed using BIA evaluation software (version 3.1), gave a K_D_ of 0.555 nM (Figure 5A). The competitive binding assay was conducted with mortalin (13 nM) pre-incubated with increasing amounts of MA or MKT077, a well-known mortalin–p53 disruptor, selected as control compound. Analysis of the corresponding sensorgrams showed a lower association of mortalin with p53, measuring strongly increased dissociation constants of 467.5 nM and 843.4 nM when mortalin was injected in the presence of MA or MKT-077, respectively (Figure 5B,C).

## 3. Discussion

Mycalin A (MA), a polybrominated C-15 acetogenin isolated from the marine sponge *Mycale rotalis*, has been identified as an interesting small molecule due to its selective cytotoxic activity against cancer cell lines, such as A375 (human melanoma) and HeLa (cervical adenocarcinoma). Functional proteomic approaches, such as DARTS and t-LiP-MS, were applied for the target deconvolution of MA.

DARTS results led to the identification of mortalin, a mitochondrial Hsp70, as one of the most reliable MA interacting partners, along with HS71A. T-LiP-MS analysis of the MA–mortalin and MA–HS71A complexes gave information on the specific protein regions involved in the binding with the small molecule. These data were corroborated by molecular docking analysis, which allowed for the evaluation of the MA best interaction poses with both mortalin and HS71A.

Mortalin is known to be overexpressed in many types of tumor tissues and shows its oncogenic functions sequestering the tumor suppressor p53. For this reason, mortalin was selected as the best MA target and a deep evaluation of their interaction was carried out. qPCR experiments were performed on p53 gene targets and showed an increased p53 transcriptional activity in responsive cancer cells treated with MA. These results confirmed that MA treatment can disrupt the mortalin–p53 complex, allowing the free p53 to exert its onco-suppressor properties. It is worth noting that the reported MA cytotoxic activity towards specific cancer cells, a very important aspect in the development of new cancer drugs, was also assessed. A complementary SPR approach gave clear evidence of the ability of MA to modulate the interaction between mortalin and its client protein p53, decreasing their binding in a similar manner to the reference compound MKT-077 (a known mortalin interactor), confirming the qPCR results.

These findings support the MA cytotoxic activity, specifically addressed to tumor cells. Mortalin is overexpressed in cancer cells, and in turn its interaction equilibrium with p53 can be more efficient in amplifying the pro-apoptotic effects of the released p53. On this basis, MA can be considered an interesting reference compound in the development of a new class of potential anticancer agents.

## 4. Materials and Methods

### 4.1. Cell Culture for DARTS Experiment

The human uterine cervical cancer cells HeLa were obtained from the American Type Culture Collection (ATCC, LGC Standards, Sesto San Giovanni, Italy) and grown (37 °C, 5% CO_2_ atmosphere) in Dulbecco’s modified Eagle medium (DMEM, Sigma Aldrich, Milan, Italy) supplemented with 10% (vol/vol) fetal bovine serum, 100 U/mL penicillin and 100 mg/mL streptomycin (reagents were from Euroclone, Milan, Italy). Cells were then harvested, collected by centrifugation (1000× *g*, 5 min) and washed three times with phosphate saline buffer (PBS: 137 mM NaCl, 2.7 mM KCl, 10 mM Na_2_HPO_4_, 2 mM KH_2_PO_4_, pH 7.4).

### 4.2. DARTS Experiment

HeLa cells proteome was extracted by mechanical lysis (Dounce homogenizer, Sigma Aldrich, Milan, Italy), suspending the pellet in ice-cooled PBS containing 0.1% Igepal and a cocktail of protease inhibitors (Sigma–Aldrich, St. Louis, MO, USA) and carrying out alternative cycles of friction and rest (4 °C). The obtained suspension was submitted to centrifugation (10,000 rpm, 4 °C, 5 min, Centrifuge 5424 R, Eppendorf, Milan, Italy) to remove the protein solution from the pelleted debris. Proteins concentration of the cleared supernatant was determined by Bradford assay (Bio-Rad, Hercules, CA, USA) and adjusted to 3 μg/μL with PBS.

Aliquots of 300 μg of the obtained proteome were incubated with either MA (0.5 μM, 5 μM or 50 μM final concentrations) or DMSO as a control (final DMSO amount: 1% vol/vol), for 1 h at room temperature and under continuous agitation. The samples were then submitted to limited proteolysis with subtilisin (subtilisin to proteins ratio of 1:750 *w*/*w*), for 30 min at 25 °C and 500 rpm. An aliquot of the DMSO-treated lysate was submitted to a mock proteolysis, adding H_2_O instead of the enzyme and treating the sample as previously reported. Subtilisin was then quenched with 1 mM final concentration of PMSF (phenylmethylsulfonyl fluoride, Sigma–Aldrich, St. Louis, MO, USA) for 10 min at 25 °C and 500 rpm. Subsequently, 20 μg of each sample were boiled in SDS-PAGE loading buffer (60 mM Tris/HCl pH 6.8, 2% SDS, 0.001% bromophenol blue, 10% glycerol, 2% 2-mercaptoethanol) and loaded on a 4–12% Bis-Tris Criterion^TM^ XT Precast Gel (BioRad Laboratoties, Hercules, CA, USA). The gel was then fixed for 15 min (fixing solution: 50% H_2_O, 40% MeOH, 10% AcOH), washed three times with H_2_O and then submitted to Coomassie staining for 1 h at room temperature and under continuous shaking. The excess dye was then removed by extensively washing the gel with H_2_O. The scan image of the resulting gel was obtained through LabScan and submitted to a densitometric analysis through ImageJ. Data were represented, as percentages of the mock-proteolyzed sample, through GraphPad Prism 7.

Gel bands corresponding to MA-protected proteins were excised and submitted to in situ tryptic digestion, as previously reported by Shevchenko et al. [23]. Briefly, they were excided form all the gel lanes and cut in smaller pieces, which were then washed by shrinking/swelling cycles using CH_3_CN and ammonium bicarbonate (AmBic, 50 mM, pH 8.5), alternatively. Then, disulfide bonds were reduced with 1,4-dithiothreitol (DTT, 6.5 mM in 50 mM AmBic, 60 min, 60 °C) and the formed thiols were carboxyamidomethylated with iodoacetamide (IAA, 54 mM in 50 mM AmBic, 30 min, room temperature, in the dark). Residual reagents were removed by shrinking/swelling cycles and gel pieces rehydrated in a 12 ng/µL trypsin/LysC solution (Promega, Madison, WI, USA) on ice for 1 h. The enzymes excess was then removed, 40 µL of 50 mM AmBic were added and the digestion was allowed to proceed overnight at 37 °C. AmBic was then collected, and peptides were extracted from the gel slices shrinking them twice with 100% CH_3_CN. All the supernatants were combined, dried out under vacuum, and dissolved in 12 µL of 10% Formic Acid (FA) for the subsequent nano-flow RP-UHPLC MS/MS analysis.

Thus, 5 µL of each peptide mixture were injected into a nano-ACQUITY UHPLC system (Waters, Milford, MA, USA). Peptides were separated on a 1.7 µm BEH C18 column (Waters) at a flow rate of 280 nL/min. Peptide elution was achieved with a linear gradient of mobile phase B from 20% to 90% over 65 min (mobile phase A: 95% H_2_O, 5% CH_3_CN, 0.1% acetic acid; mobile phase B: 95% CH_3_CN, 5% H_2_O, 0.1% acetic acid). MS and MS/MS data were acquired on an Orbitrap LTQ XL high-performance liquid chromatography MS system (Thermo-Scientific, Waltham, MA, USA) equipped with an electrospray (ESI) source. The ten most intense doubly and triply charged peptide ions were chosen and fragmented.

The resulting MS data were processed through the MS Converter General User Interface software (ProteoWizard 3.0.9740 64 bit) to generate peak lists for proteins identification. Database searches were carried out on the Mascot Deamon version 5.1 by Matrix Science (London, UK), employing the Swiss Prot database (release October 2019, 95,934 sequences, 38,078,700 residues) and the following settings: two missed cleavages; carbamidomethyl (C) as fixed modification and oxidation (M) and phosphorylation (ST) as variable modifications; peptide tolerance 30 ppm; MS/MS tolerance 0.8 Da. The obtained data were filtered by molecular weight ranges accordingly with the gel cutting patterns and a semi-quantitative analysis was then performed comparing Mascot matches among the analyzed samples. Protection percentages were thus calculated, for each MA amount, as follows:

Protection (%) = [(Matches_MA_ − Matches_Control_)/Matches_Lysate_] × 100.

The above-described experiment was carried out in duplicate.

### 4.3. Western Blotting

30 μg of the DARTS obtained protein mixtures were boiled in SDS-PAGE loading buffer and loaded on a 12% SDS-PAGE to be transferred onto nitrocellulose membranes. The membranes were blocked, for 1 h at room temperature, in a TBS-t solution (30 mM Tris pH 8, 170 mM NaCl, 3.35 mM KCl, 0.05% vol/vol Tween-20) containing 5% w/vol non-fat dried milk and then incubated overnight at 4 °C with primary monoclonal antibodies raised against either mortalin or HS71A (1:1000 vol/vol, Santa Cruz Biotechnology, Inc., Dallas, TX, USA). The antibodies excess was then removed, membranes were washed three times with TBS-t and incubated, for 1 h at room temperature, with a mouse peroxidase-conjugated secondary antibody (1:2500 vol/vol; Thermo-Scientific, Waltham, MA, USA). The signal was detected using an enhanced chemiluminescent substrate and LAS 4000 (GE Healthcare, Waukesha, WI, USA) digital imaging system. Afterwards, the membranes were also hybridized with an anti-Glyceraldehyde 3-Phosphate Dehydrogenase antibody (GAPDH, 1:2000 vol/vol, mouse, Invitrogen, Carlsbad, CA, USA) and the signal was detected as already described. Then, a densitometric analysis was performed through ImageJ: data were elaborated using GAPDH as a loading normalizer and rating undigested mortalin/HS71A intensity as 100%. The obtained values were represented through GraphPad Prism 7.

### 4.4. T-LiP-MS Analysis

Mortalin (UniProt accession number: P38646) and HS71A (UniProt accession number: P0DMV8) tryptic peptides previously detected by MS were selected through the proteomics data resource Peptide Atlas (https://db.systemsbiology.net/sbeams/cgi/PeptideAtlas/Search, accessed on 20 October 2019 on its Human build and queried into the complete Human SRM Atlas build (https://db.systemsbiology.net/sbeams/cgi/PeptideAtlas/GetTransitions, accessed on 15 October 2023), to retrieve their best daughter ions. The SRM Atlas query parameters were as follows: number of highest intensity fragment ions to keep: 8; target instrument: QTRAP 5500; transitions source: QTOF, Agilent QQQ, Qtrap5500, Ion Trap, predicted; search proteins form: Swiss Prot; duplicate peptides: unique in results; allowed ions types: b-ions and y-ions; allowed peptides modification: carbamidomethylation of cysteines. The obtained transitions lists, containing 35 precursors and 280 fragmentations for mortalin and 26 precursors and 208 fragment ions for HS71A, were subsequently refined as follows: peptides whose following C-terminus amino acid was either K or R were removed, as well as peptides whose *m/z* ratio was higher than 1100 Da; among the 8 fragment ions reported for each precursor, only the three most intense y-series ones were selected.

Thus, methods listing 26 and 17 peptides for mortalin and HS71A respectively, each with their three best transitions, were obtained and subsequently tested onto a HeLa lysate tryptic digest.

For this purpose, 300 µg of HeLa cell lysate obtained as described before were submitted to an in-solution digestion protocol. Briefly, proteins were denatured using 8 M urea/50 mM AmBic (4 M final urea concentration), disulfide bonds were reduced with 10 mM DTT (1 h, 25 °C and 800 rpm) and then alkylated with 20 mM IAA (30 min in the dark, 25 °C and 800 rpm). IAA was then quenched with 10 mM DTT (10 min, 25 °C, 800 rpm) and urea was diluted up to 1 M with 50 mM AmBic before adding a trypsin/LysC solution (Promega, Madison, WI, USA) at the enzymes to proteins ratio of 1:100 *w*/*w*. Digestion was allowed to proceed overnight at 37 °C and 800 rpm and then quenched adding FA to lower the pH to 3. Peptides mixture was then dried under vacuum, dissolved in 1 mL 5% FA and desalted through a Sep-Pak C18 1 cc (50 mg) cartridge (Waters, Milford, MA, USA). The cartridge was activated flushing 3 mL of 100% CH_3_CN and then conditioned with 3 mL of 0.1% FA. The sample was then loaded, desalted flushing the cartridge with 3 mL of 0.1% FA and finally eluted flushing two times 500 μL of 80% CH_3_CN, 20% H_2_O, 0.1% FA. For the subsequent MS analysis, the peptides mixture was dried under vacuum and re-dissolved in 10% FA.

UHPLC–ESI-MRM-MS analyses were performed on a 6500 Q-TRAP from AB Sciex equipped with Shimadzu LC-20A and Auto Sampler systems. UHPLC separation was performed on an Aeris Widepore XB C18 column (150 × 2.10 mm, 3.6 μm XB, Phenomenex, Torrance, CA, USA), using 0.1% FA in H_2_O (A) and 0.1% FA in CH_3_CN (B) as mobile phases, and a linear gradient from 5 to 95% of B over 30 min (flow rate: 200 μL/min). Q-TRAP 6500 was operated in positive MRM scanning mode, with declustering potential (DP) set at 80 V, entrance potential (EP) at 10 V, collision energy (CE) at 35 V and cell exit potential (CXP) at 22 V. UHPLC–ESI-MRM/MS runs were performed injecting 15 µg of the peptides mixture: the XICs of all the transitions of each precursor were inspected to assign the retention time to all of the peptides and to identify their best transition, as the one showing the most intense peak and the best signal to noise ratio. Thus, a global MRM method comprising 20 transitions for mortalin and 11 transitions for HS71A was obtained.

Then, 300 μg of HeLa cells proteome obtained as previously reported were incubated with DMSO or MA (50 μM final concentration, final DMSO amount 1% vol/vol), for 1 h at room temperature and under continuous agitation. The samples were then submitted to limited proteolysis (1:1500 *w*/*w* subtilisin to proteins ratio), for 30 min at 25 °C and 500 rpm, keeping 300 μg of the DMSO-treated lysate undigested as a positive control. Subtilisin was then quenched with PMSF (1 mM final concentration) and the samples shifted to denaturing condition adding urea (4 M final concentration) to perform in-solution digestion and desalting, as described before.

The obtained peptides mixtures were dissolved in 10% FA and 15 μg were injected in the UHPLC–ESI-MRM-MS system and analyzed through the previously optimized MRM methods. Three injection replicates were performed. The area of each mortalin and HS71A tryptic peptide peak was then measured using the Analyst Software (version 1.6.2) from AB Sciex. Data analysis was performed through GraphPad Prism 7: results were represented as fold changes (Area_MA_/Area_CTRL_) with the corresponding *p*-values. MA-protected peptides were mapped, through SPDB viewer, onto the following PDB structures: pdb ID 2e88 [30] for HS71A and 4kbo [27] and 3n8e [31] for mortalin.

### 4.5. Interactions of the Complexes Mycalin A/Mortalin and Mycalin A/HS71A: Computational Studies

The prediction of the binding between MA and human mortalin and HS71A was carried out by performing a molecular docking analysis, which consisted of the ligand and protein preparation, the execution of the genetic algorithm (GA) execution and the data analysis and images preparation. The ligands were designed, including the addition of tautomeric states, partial charges and protonation, and finally minimized using the Avogadro software (version 1.2.0) [32] with a universal force field, UFF, and a conjugate gradient algorithm until a ΔE lower than 0.001 kJ/mol, as previously reported [33]. The protein three-dimensional structures were obtained from the Protein Data Bank [34] (HS71A pdbIDs: 2e88 [30] and 5gjj20; mortalin pdbIDs: 4kbo [27] and 3n8e [31]) and prepared, using the Hermes software (version 1.10.0) 15 incorporating the Gasteiger (-Marsili) partial charges, adding polar protons and removing crystal waters and extra co-crystallized ligands. Moreover, all planar R-NR1R2 were made available for the cis/trans flipping and the tautomeric states of Asp, Glu and His residues. GOLD (version 5.7.0) [35] was employed for molecular docking, utilizing ChemScore as the scoring function. This function comprises protein-ligand hydrogen bond energy (external H-bond), protein-ligand VdW energy (external), ligand internal VdW energy and ligand torsional strain energy (internal torsion) in the docking process. The search efficiency was at 200% (very flexible), selecting all atoms within 20 Å from the centroid (covering almost completely the whole protein), 20 GA runs and other parameters as default. The resulting Chem Score Δ*G*, the total free energy change of the system upon ligand binding, and the relationship between this score and experimental free energy of binding, previously obtained [36], were used to calculate the predicted equilibrium dissociation constant K_D,pred_. The best complex geometry, determined based on the Chem Score and Chem Score Δ*G*, was rendered using the PyMol software (The PyMOL Molecular Graphics System, Version 2.0.4 Schrödinger, LLC.). Additionally, the 3D representation of the interaction was generated using the Protein–Ligand Interaction Profiler (PLIP) web server [37].

### 4.6. Biological Assays

Human cervical carcinoma HeLa, human epithelial melanoma A375 and human breast carcinoma MCF7 cells were cultured in DMEM, with high glucose, containing 10% bovine fetal serum (Euroclone, Pero, MI, Italy), 2 mM L-glutamine, 100 μg/mL penicillin and 100 μg/mL streptomycin, in a humidified incubator at 5% CO_2_ at 37 °C [38]. A total of 1.5 × 10^6^ cells were plated in 10 cm culture dish incubated at 37 °C for 24 h. MR1 at a concentration of 2 µM was added to the cells and the lysis was performed after 24 h of treatment.

Total RNA was obtained from cells after 2 µM MR1 treatment, using TRI Reagent (Sigma Aldrich) according to manufacturing instructions. Reverse transcription was performed using Lunascript supermix kit, setting the reaction temperature at 60 °C for 10 min. After that, qPCR assay was performed using the following primers:

GAPDH forward 5′-AACGGGAAGCTTGTCATCAATGGAAA-3′

GAPDH reverse 5′-GCATCAGCAGAGGGGGCAGAG-3′

p21-forward:5′-GGAGACTCTCAGGGTCGAAAACG-3′

p21-reverse: 5′-CGGATTAGGGCTTCCTCTTGGAG-3′

puma-forward: 5′-ACGACCTCAACGCACAGTACGAG-3′,

puma-reverse: 5′-GTAAGGGCAGGAGTCCCATGATG-3′;

mdm2-forward: 5′-GCAGGGGAGAGTGATACAGATTC-3′,

mdm2-reverse: 5′-AATGTGATGGAAGGGGGGGATTC-3′;

mortalin-forward: 5′-AGCTGGAATGGCCTTAGTCAT-3′,

mortalin-reverse: 5′-CAGGAGTTGGTAGTACCCAAA-3′;

(IDT, Coralville, IA, USA). Quantitative PCR (qPCR) amplification reactions were carried out using the SYBR Premix Ex Taq II (Takara, Shiga, Japan) in Rotor-gene Q (Qiagen, Milan, Italy) and performed in duplicate. The qPCR protocol was as follows: 95 °C for 15 min followed by 40 cycles of 95 °C for 15 s, 59 °C for 30 s, and 72 °C for 30 s. The results, expressed as relative fold induction of each target genes respect to the reference gene, were obtained by calculating the relative expression levels using the 2-DDCt method [39] and averaging the values of at least three independent experiments.

### 4.7. Surface Plasmon Resonance (SPR)

Full-length p53 (cat. No. 230-00639) was purchased from RayBiotech (Peachtree Corners, GA, USA). Series S Sensor Chip CM5 (cat. no. BR100530), Amine Coupling Kit (cat. no. BR100050), HBS-P (cat. no. BR100368) were purchased from Cytiva (Uppsala, Sweden).

SPR analyses were performed using a Biacore T200 (Cytiva, Uppsala, Sweden) optical biosensor equipped with research-grade CM5 (Carboxy Methyl Dextran) sensor chip.

#### Direct and Competition Binding Assays

The affinity of mortalin for p53 protein was determined. Prior to the immobilization of the p53 protein, a pH scouting was performed as follows. Solutions of 0.86 µM of the ligand in 10 mM sodium acetate with pH values ranging from 4.61 to 6 were prepared and injected onto the surface [40,41].

The p53 protein (0.86 µM in 10 mM sodium acetate, pH 4.61) was immobilized by using standard amine-coupling protocol to obtain densities of 7700 RU. HBS-P buffer (0.01 M HEPES pH 7.4, 0.15 M NaCl, 0.005% (*v*/*v*) Surfactant P20), supplemented with 2% DMSO was used as a running buffer. Stock solution of mortalin in 100% DMSO was prepared (10 mM). Running buffer was injected at a flow rate of 30 µL/min over the chip to clean and equilibrate the immobilizes sensor surface, then a solvent correction was performed as indicated by Biacore application guides (Cytiva). A series of increasing concentrations of mortalin (0.22–7 nM) diluted in the ligand buffer were injected at 25 °C with a flow rate of 10 µL/min for 90s (association phase), and then the buffer alone was injected for 400 s (dissociation phase). A regeneration step was not necessary. The first channel was used as a reference surface. All mathematical manipulations and fitting operations were performed using BIAevaluation software (v3.1) provided with the Biacore T200 instrument (Cytiva) and assuming a 1:1 Langmuir binding model.

The competitive binding assay was conducted in HBS-P (pH 7.4) at 25 °C. Mortalin (13 nM) was pre-incubated with increasing amounts of MA or MKT077 (0.026–0.85 µM) for 60 min before SPR analysis. The mixture solution containing mortalin and different concentrations of MA or MKT077 was injected over p53-immobilized sensor chips at a flow rate of 10 µL/min. Fitting operations were performed as described above.

## Figures and Tables

**Table 1:** qPCR analysis of mRNA levels of indicated genes in A375 and MCF7 cells treated with MA. Results are presented as fold change in gene expression relative to the reference gene (GAPDH). Data were analyzed using the 2-ΔΔCt method. 24 SD = standard deviation. CV = percent coefficient of variation of 2-ΔΔCt.

	A375			MCF7		
	Mean	SD	CV	Mean	SD	CV
**Control**	1.00	0.00	0.00	1.00	0.00	0.00
**P21**	1.46	0.07	4.80	0.94	0.25	26.47
**puma**	1.38	0.04	2.60	0.90	0.11	12.57
**Mdm2**	1.79	0.28	15.80	0.93	0.04	3.82
**Grp75**	1.10	0.05	4.50	0.92	0.10	11.59

## Data Availability

All data are contained into the article and Supplementary Materials.

## Supplementary Materials

The following supporting information can be downloaded at: https://www.mdpi.com/article/10.3390/md22020052/s1, Figure S1: Densitometric analysis of the Western Blots analysis of DARTS experiments; Figure S2: mortalin and HS71A fully tryptic peptides areas in the t-LiP experiment; Figure S3: Sticks and ribbons representation of mortalin NBD and SBD and of HS71A NBD; Table S1: List of proteins identified in two independent DARTS experiments, reported with their Mascot Score and Matches; Table S2: Mortalin and HS71A fully tryptic peptides analyzed through t-LiP-MS.
