# Supplementary material for: Identification of Mortalin as the Main Interactor of Mycalin A, a Poly-Brominated C-15 Acetogenin Sponge Metabolite, by MS-Based Proteomics
Source: Mar Drugs. 2024 Jan 23;22(2):52. doi: 10.3390/md22020052 (PMC10890321; doi:10.3390/md22020052)
Supplement: Supplementary file 1 [file marinedrugs-22-00052-s001.zip › marinedrugs-2788383-supplementary.pdf]

Identification of Mortalin as the Main Interactor of Mycalin A, a Poly-Brominated C-15 Acetogenin Sponge Metabolite, by MS-Based Proteomics

Elva Morretta [1,†], Alessandra Capuano [1,2,†], Gilda D'Urso [1], Antonia Voli [1,2], Matteo Mozzicafreddo [3], Sonia Di Gaetano [4], Domenica Capasso [5], Marina Sala [1], Maria Carmina Scala [1], Pietro Campiglia [1], Vincenzo Piccialli [6,*] and Agostino Casapullo [1,*]

[1] Department of Pharmacy, University of Salerno, 84084 Fisciano, Italy; emorretta@unisa.it (E.M.); acapuano@unisa.it (A.C.); gidurso@unisa.it (G.D.); avoli@unisa.it (A.V.); msala@unisa.it (M.S.); mscala@unisa.it (M.C.S.); pcampiglia@unisa.it (P.C.)
[2] PhD Program in Drug Discovery and Development, University of Salerno, 84084 Fisciano, Italy
[3] Department of Clinical and Molecular Sciences, Marche Polytechnic University, 60126 Ancona, Italy; m.mozzicafreddo@univpm.it (M.M.)
[4] Institute of Biostructures and Bioimaging, Consiglio Nazionale delle Ricerche, Via Pietro Castellino 111, 80131 Napoli, Italy; sonia.digaetano@cnr.it (S.D.G.)
[5] Department of Physics, Ettore Pancini, University of Naples "Federico II", Via Cintia 21, 80126 Naples, Italy; domenica.capasso@unina.it (D.C.)
[6] Department of Chemical Sciences, University of Naples "Federico II,", Via Cintia 21, 80126 Naples, Italy
* Correspondence: vinpicci@unina.it (V.P.); casapullo@unisa.it (A.C.)
† These authors contributed equally to this work.

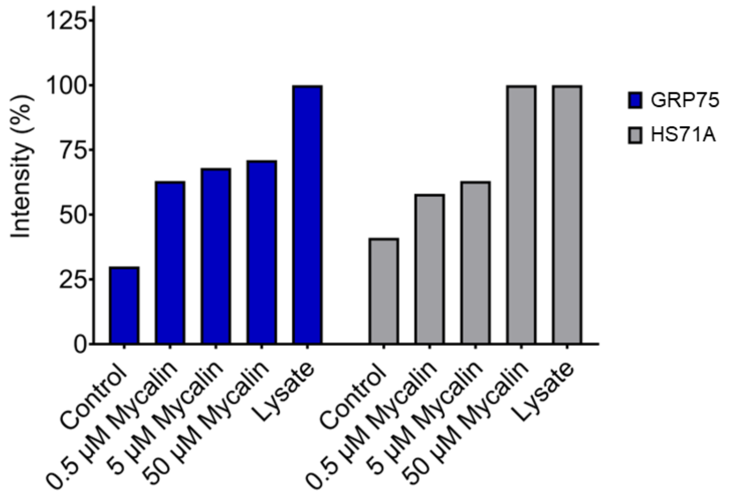

Figure S1 Densitometric analysis of the Western Blots analysis of DARTS experiments

Densitometric analysis of the Western Blots experiments was performed through ImageJ on the full-length GRP75 signals (blue bars) and on the HS71A ones (gray bars). Increasing MA concentrations protect both Hsp70 isoforms accordingly.

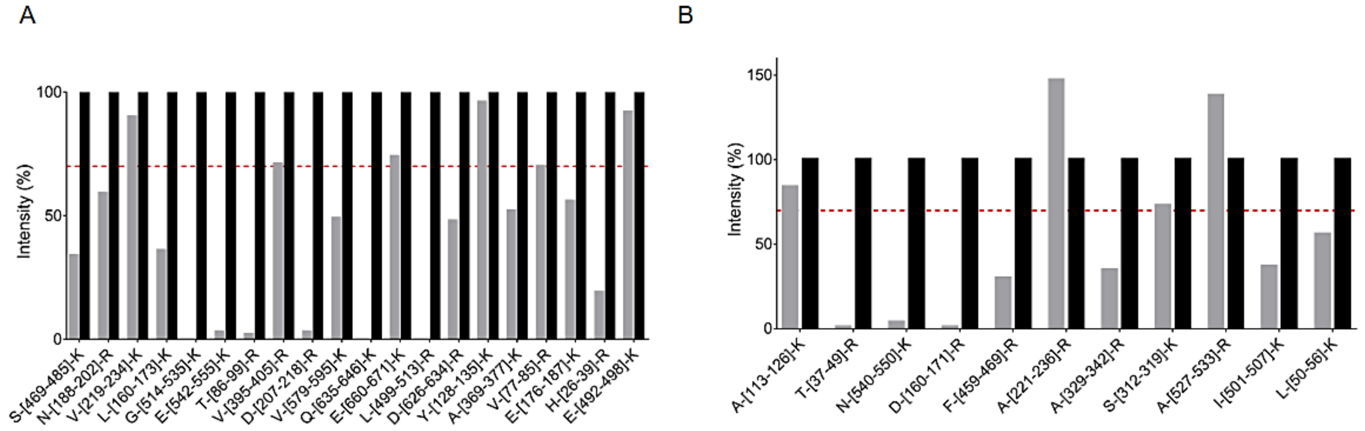

Figure S2 GRP75 and HS71A fully tryptic peptides areas in the t-LiP experiment

GRP75 (A) and HS71A (B) fully tryptic peptides areas of the negative (i.e., DMSO and subtilisin treated sample, gray bars) and positive (i.e., DMSO treated and subtilisin unexposed sample, black bars) controls, reported as percentages of the latter sample.

Negative control peptides whose intensity is higher than the 70% of the corresponding positive control ones are those whose (gray) bars exceed the red dashed lines in both graphs. Mapping for GRP75 and HS71A regions not affected by subtilisin, these peptides were not considered for the subsequent data analysis step.

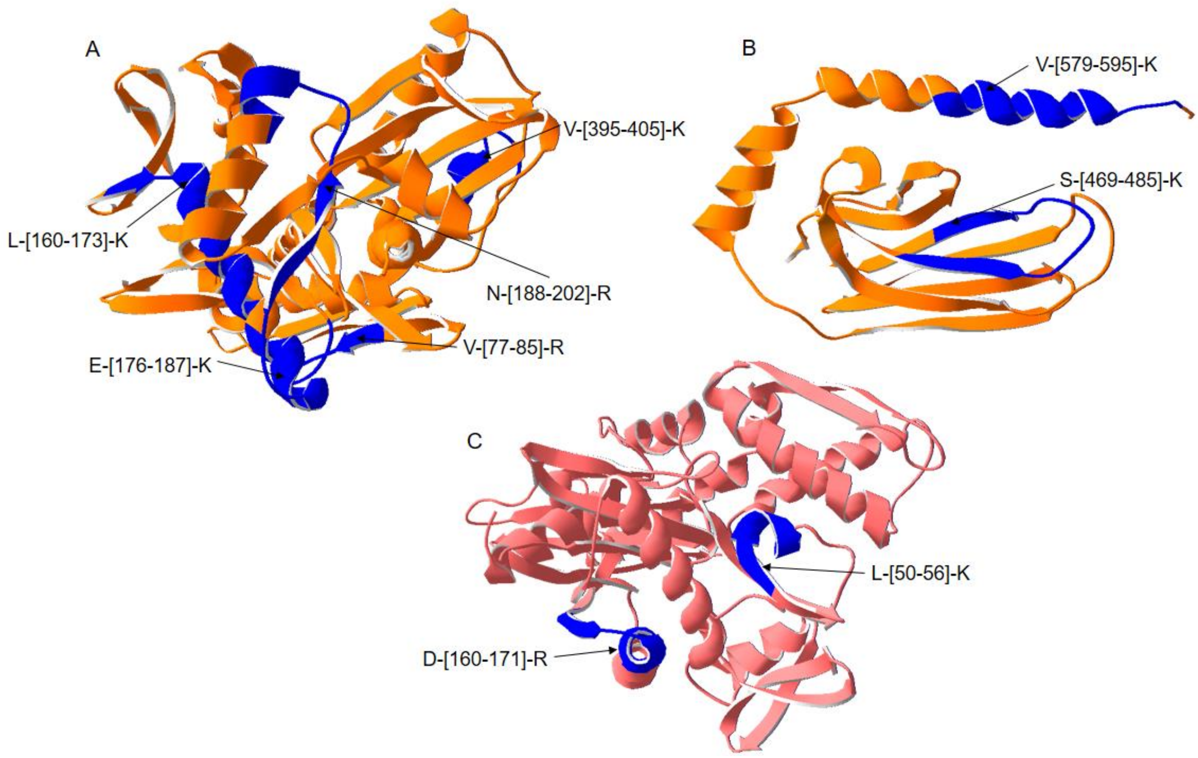

Figure S3 Sticks and ribbons representation of GRP75 NBD and SBD and of HS71A NBD

Sticks and ribbons representation of GRP75 NBD (A) and SBD (B) and of HS71A NBD (C). MA protected peptides identified through t-LiP-MS are depicted in blue and indicated with the corresponding identifiers. As can be observed in panels A and B, GRP75 peptides all map for the same NBD and SBD regions, suggesting an important conformational change following MA binding and, therefore, a minor sensitivity to subtilisin proteolytic action. The only exception to this trend is the NBD V-[395-405]-K, which lies far from the other peptides.

Regarding HS71A NBD, as can be observed in panel C, the two identified protected peptides do not seem to be close to one another, but they face the same α-helix.

Table S1 List of proteins identified in two independent DARTS experiments, reported with their Mascot Score and Matches.

			DARTS 1										DARTS 2									
			Control		0.5 μM MA		5 μM MA		50 μM MA		Lysate		Control		0.5 μM MA		5 μM MA		50 μM MA		Lysate	
Accession	Mass	Description	Score	Matches	Score	Matches	Score	Matches	Score	Matches	Score	Matches	Score	Matches	Score	Matches	Score	Matches	Score	Matches	Score	Matches
P17655	80 800	Calpain-2 catalytic subunit	0	0	0	0	0	0	0	0	96	4	0	0	0	0	0	0	106	5	203	6
P54136	76 129	Arginine--tRNA ligase, cytoplasmic	0	0	33	2	0	0	68	1	226	4	310	8	311	6	392	10	529	17	786	22
**P38646**	**73 920**	**Stress-70 protein, mitochondrial**	**465**	**10**	**702**	**16**	**773**	**21**	**1281**	**25**	**1147**	**32**	**820**	**25**	**1167**	**34**	**1289**	**35**	**1519**	**44**	**1977**	**53**
P11142	71 082	Heat shock cognate 71 kDa protein	2973	84	3424	107	2723	75	1920	44	3350	97	4074	119	3435	99	4489	131	5868	163	6375	181
**P0DMV8**	**70 294**	**Heat shock 70 kDa protein 1A**	**0**	**0**	**0**	**0**	**0**	**0**	**58**	**2**	**430**	**9**	**530**	**15**	**705**	**21**	**1066**	**29**	**1423**	**43**	**1270**	**47**
P12956	70 084	X-ray repair cross-complementing protein 6	0	0	256	17	111	11	0	0	486	29	1442	60	2013	81	1770	78	1907	79	2266	91
O60506	69 788	Heterogeneous nuclear ribonucleoprotein Q	114	2	171	3	385	9	0	0	195	5	0	0	0	0	0	0	0	0	97	4
P15311	69 484	Ezrin	119	4	0	0	48	3	0	0	296	15	0	0	215	14	0	0	452	23	1098	41
P29401	68 519	Transketolase	334	5	0	0	361	8	565	13	1815	51	3061	89	4435	122	3655	116	4324	151	3977	112
Q15046	68 461	Lysine--tRNA ligase	0	0	0	0	0	0	0	0	157	6	0	0	0	0	0	0	114	3	385	15
P26038	67 892	Moesin	0	0	194	7	0	0	0	0	646	24	98	3	423	16	230	11	546	23	1426	56
P17987	60 819	T-complex protein 1 subunit alpha	33	2	0	0	0	0	0	0	129	6	0	0	78	5	498	20	30	5	654	23
P48643	60 089	T-complex protein 1 subunit epsilon	286	6	324	4	179	8	0	0	837	14	0	0	901	22	1350	41	614	20	1141	37
Q99832	59 842	T-complex protein 1 subunit eta	0	0	0	0	0	0	0	0	691	12	0	0	471	11	911	26	119	3	1373	39
P25705	59 828	ATP synthase subunit alpha, mitochondrial	230	5	68	1	320	6	0	0	419	11	0	0	0	0	0	0	128	5	363	11
P11413	59 675	Glucose-6-phosphate 1-dehydrogenase	0	0	0	0	81	5	0	0	156	9	0	0	0	0	0	0	0	0	18	3
P40227	58 444	T-complex protein 1 subunit zeta	185	14	82	9	113	6	114	6	1067	23	318	10	817	24	1533	43	1039	34	1808	58
P50991	58 401	T-complex protein 1 subunit delta	191	9	0	0	0	0	0	0	701	25	0	0	231	12	513	20	0	0	1211	41
P78371	57 794	T-complex protein 1 subunit beta	123	5	68	1	0	0	0	0	1069	22	0	0	93	1	586	15	0	0	1026	30
O43175	57 356	D-3-phosphoglycerate dehydrogenase	479	13	269	9	415	13	101	8	567	18	0	0	0	0	532	17	529	15	812	24
P23381	53 474	Tryptophan--tRNA ligase, cytoplasmic	0	0	0	0	0	0	0	0	445	13	0	0	0	0	14	2	56	1	791	19
Q01518	52 325	Adenylyl cyclase-associated protein 1	100	4	0	0	0	0	0	0	444	17	0	0	0	0	259	12	0	0	956	34
P55209	45 631	Nucleosome assembly protein 1-like 1	49	4	0	0	146	5	0	0	357	11	0	0	0	0	31	1	0	0	369	11

The list was obtained removing subtilisin-undigested proteins (i.e., $Matches_{Control} \geq Matches_{Lysate}$) and proteins that were not identified in MA-treated samples in both DARTS replicates. As can be observed, the mitochondrial stress-70 protein (referred to as GRP75 in the main text) and the cytosolic Heat shock 70 kDa protein A1 (referred to as HS71A in the main text) score and matches increase when increasing MA amounts were incubated with the HeLa lysates prior to subtilisin limited proteolysis. Control: DMSO and subtilisin treated sample, or negative control in the main text. Lysate: DMSO-treated and undigested sample or positive control in the main text.

Table S2 GRP75 and HS71A fully tryptic peptides analyzed through t-LiP-MS

			GRP75		HS71A	
Q1_m/z	Q3_m/z	Retention Time (min)	Sequence	Peptide	Sequence	Peptide
421.7	322.19	4.86	HQDSWNGLSHEAFR	H-[26-39]-R	-	-
479.75	746.34	2.61	VLENAEGAR	V-[77-85]-R	-	-
725.86	866.4	8.2	TTPSVVAFTADGER	T-[86-99]-R	-	-
~~497.23~~	~~715.36~~	~~21.81~~	~~YDDPEVQK~~	~~Y-[128-135]-K~~	-	-
777.42	278.15	11.71	LYSPSQIGAFVLMK	L-[160-173]-K	-	-
445.22	513.28	5.63	ETAENYLGHTAK	E-[176-187]-K	-	-
847.93	1097.5	9.65	NAVITVPAYFNDSQR	N-[188-202]-R	-	-
621.84	758.45	8.93	DAGQISGLNVLR	D-[207-218]-R	-	-
~~823.44~~	~~666.35~~	~~22.28~~	~~VINEPTAAALAYGLDK~~	~~V-[219-234]-K~~	-	-
489.73	234.15	22.15	AMQDAEVSK	A-[369-377]-K	-	-
645.84	1063.55	8.66	VQQTVQDLFGR	V-[395-405]-R	-	-
904.96	902.49	7.51	SQVFSTAADGQTQVEIK	S-[469-485]-K	-	-
~~382.67~~	~~261.16~~	~~5.02~~	~~EMAGDNK~~	~~E-[492-498]-K~~	-	-
531.66	269.16	12.23	LLGQFTLIGIPPAPR	L-[499-513]-R	-	-
770.41	305.18	9.98	GVPQIEVTFDIDANGIVHVSAK	G-[514-535]-K	-	-
737.4	876.48	9.56	EQQIVIQSSGGLSK	E-[542-555]-K	-	-
619.64	672.83	10.3	VEAVNMAEGIIHDTETK	V-[579-595]-K	-	-
510.73	689.36	5.48	DSETGENIR	D-[626-634]-R	-	-
616.34	674.38	10.89	QAASSLQQASLK	Q-[635-646]-K	-	-
562.25	275.17	21.96	EGSGSSGTGEQK	E-[660-671]-K	-	-
744.35	643.31	8.02	-	-	TTPSYVAFTDTER	T-[37-49]-R
344.21	218.15	1.3	-	-	LIGDAAK	L-[50-56]-K
807.91	248.16	22.35	-	-	AFYPEEISSMVLTK	A-[113-126]-K
599.35	742.46	9.56	-	-	DAGVIAGLNVLR	D-[160-171]-R
~~559.25~~	~~272.17~~	~~1.65~~	-	-	~~ATAGDTHLGGEDFDNR~~	~~A-[221-236]-R~~
451.75	375.22	7.29	-	-	STLEPVEK	S-[312-319]-K
489.28	288.66	4.78	-	-	AQIHDLVLVGGSTR	A-[329-342]-R
~~592.33~~	~~537.31~~	~~9.07~~	-	-	~~FELSGIPPAPR~~	~~F-[459-469]-R~~
402.73	376.18	5.85	-	-	ITITNDK	I-[501-507]-K
~~423.7~~	~~201.63~~	~~6.24~~	-	-	~~AEDEVQR~~	~~A-[527-533]-R~~
644.31	989.44	9.2	-	-	NALESYAFNMK	N-[540-550]-K

Each peptide is reported with its precursor m/z value (i.e., Q1_m/z), its best daughter ion m/z value (i.e., Q3_m/z) and the corresponding retention time, sequence, and identifier (i.e., *Peptide* columns in the table). Q3_m/z and retention times were assigned analyzing a HeLa tryptic digest through previous MRM methods consisting of three transitions for each precursor. Thus, the extracted ion currents (i.e., XICs) of all the transitions for each precursor were inspected to assign them the corresponding retention times (same precursor = same retention time for all the daughter ions) and subsequently select the best daughter ion as the one giving the highest signal and the lowest noise level.

Crossed rows indicate peptides whose area in the negative control was higher than the 70% of the corresponding positive control one, as reported in the subsequent figure.
